# Modeling Cell-Cell Interactions in Parkinson’s Disease Using Human Stem Cell-Based Models

**DOI:** 10.3389/fncel.2019.00571

**Published:** 2020-01-17

**Authors:** Katrin Simmnacher, Jonas Lanfer, Tania Rizo, Johanna Kaindl, Beate Winner

**Affiliations:** Department of Stem Cell Biology, Friedrich-Alexander-Universität Erlangen-Nürnberg, Erlangen, Germany

**Keywords:** Parkinson’s disease, iPSC, neurodegeneration, organoid, dopaminergic neuron, disease modeling, inflammation, glia

## Abstract

Parkinson’s disease (PD) is the most frequently occurring movement disorder, with an increasing incidence due to an aging population. For many years, the post-mortem brain was regarded as the gold standard for the analysis of the human pathology of this disease. However, modern stem cell technologies, including the analysis of patient-specific neurons and glial cells, have opened up new avenues for dissecting the pathologic mechanisms of PD. Most data on morphological changes, such as cell death or changes in neurite complexity, or functional deficits were acquired in 2D and few in 3D models. This review will examine the prerequisites for human disease modeling in PD, covering the generation of midbrain neurons, 3D organoid midbrain models, the selection of controls including genetically engineered lines, and the study of cell-cell interactions. We will present major disease phenotypes in human *in vitro* models of PD, focusing on those phenotypes that have been detected in genetic and sporadic PD models. An additional point covered in this review will be the use of induced pluripotent stem cell (iPSC)-derived technologies to model cell-cell interactions in PD.

## Introduction Parkinson’s Disease

Parkinson’s disease (PD) is the second most frequently occurring neurodegenerative disorder. Clinically, PD patients suffer from motor symptoms, such as bradykinesia, rigor, and tremor, with varying degrees of severity (Postuma et al., 2015). Neuropathological hallmarks of PD include the selective loss of midbrain dopaminergic (mDA) neurons in the pars compacta of the substantia nigra (SNpc), and the presence of α-synuclein-protein (α-Syn) in intracytoplasmic and intraneuritic eosinophilic inclusions, termed Lewy bodies and Lewy neurites, respectively (Pakkenberg et al., 1991; Braak and Braak, 2000). Around 90% of PD patients are diagnosed with sporadic PD, which has a largely unknown etiology. In attempting to decipher this complex etiology, environmental toxins such as heavy metals and pesticides, as well as psychostimulants, have gained increasing attention, alongside multifactorial genetic processes (Ball et al., 2019). To date, the majority of research has focused on familial PD, despite the fact that this constitutes less than 10% of all PD cases. Pathogenic variants in genes including *SNCA*, *LRRK2*, *PINK1*, and *PRKN2*, as well as rare mutations in e.g., *DJ-1*, *ATP13A2*, *PLA2G6*, and *GBA*, have been identified as critical for this disease (Singleton et al., 2013).

The gold-standard treatment for motor symptoms in PD is the pharmacological replacement of dopamine in the brain, either by increasing dopamine concentrations or by stimulating dopamine receptors (Connolly and Lang, 2014). In most PD cases, dopaminergic (DA) therapy results in a significant amelioration of motor symptoms, though this is merely symptomatic and may lose efficiency over time (Kalia and Lang, 2015). Surgical procedures, such as deep-brain stimulation, offer additional symptomatic benefits and in particular have been reported to improve on-off periods of motor symptoms observed at later stages of the disease (Limousin et al., 1998; Castrioto et al., 2014). However, no existing therapies target the neurodegenerative process itself. Furthermore, it is becoming increasingly evident that neuronal loss in PD does not arise solely from a neuron-intrinsic degenerative mechanism, but is rather the result of a complex process involving neurons and other CNS-resident and non-resident cell types (Hirsch et al., 2013).

Due to difficulties in accessing patient brain tissue, the majority of knowledge regarding PD pathology was originally obtained from the study of postmortem brains (Baba et al., 1998; Walker et al., 1998; Hunot et al., 1999). However, the development of human stem cell-based technologies has provided new possibilities for furthering our understanding of PD pathophysiology and developing new therapeutic strategies. Replacement strategies involving the transplantation of dopamine-producing human stem cells into PD patients’ striatum have been implemented and are currently evaluated by a worldwide consortium. Clinical studies are evaluating the potential of this method to improve motor symptoms while reducing or eliminating the need for DA medication (Barker et al., 2015; Studer et al., 2019). Moreover, human stem cell-based disease modeling provides a means of studying both genetic and sporadic PD and also of testing for suitable compounds that may target the neurodegenerative process in PD (Marchetto et al., 2010; Durnaoglu et al., 2011). The ongoing optimization of stem cell differentiation protocols for the generation of various cell types and multicellular structures paves the way for PD disease modeling in more complex intercellular settings (Lancaster et al., 2013; Smits et al., 2019). In this review article, we will first address the pre-requisites of stem cell-based PD research: the efficient generation of relevant models of functional mDA neurons in 2D and 3D settings. We will then provide an overview of recent stem cell-based findings in genetic and sporadic PD and describe both two- and three- dimensional approaches to the disease modeling of various cell-cell interactions.

## Human Stem Cell-Derived Models for PD Modeling

Recent advances in stem cell biology have established technologies to reprogram somatic cells to a state of pluripotency comparable to human embryonic stem cells (hESCs). The first reprogrammed cells termed induced pluripotent stem cells (iPSCs), were generated by retroviral expression of four transcription factors *Oct3/4, Sox2, Klf4*, and *c-myc (or alternatively Nanog)* from adult fibroblasts jump-starting their continuous expression (Takahashi et al., 2007). The resulting possibility to differentiate these iPSCs further into neurons of various neurotransmitter phenotypes opens new horizons for the study of CNS diseases, where human brain tissue is otherwise difficult to approach (Tao and Zhang, 2016). Alternative resources for human disease models include ESCs derived from the blastocyst, which are also able to generate a source for brain cells.

Initial midbrain differentiation protocols mimicked embryonic development by the formation of embryoid bodies or the use of undefined co-culture systems (Kawasaki et al., 2000; Perrier et al., 2004). The Studer lab later pioneered the conversion of human pluripotent cells into a primitive neuroectoderm by inhibiting the TGFβ/activin/nodal and BMP pathways, both of which signal *via* SMAD2/3 and SMAD1/5 (Heldin et al., 1997; Bond et al., 2012). This dual SMAD inhibition method was further refined by adding sonic hedgehog (Shh) pathway agonists for anterior floor plate identity and appropriately activating the WNT signaling pathway [e.g., using the GSK3β inhibitor Chiron (CHIR99021)] resulting in a majority of TH-positive floor plate derived neurons (Chambers et al., 2009; Kriks et al., 2011).

In addition to the advances made in differentiating DA neurons, the differentiation of other CNS resident cell types from iPSCs and ESCs have made considerable progress in recent years. Protocols for the differentiation of iPSC derived astrocytes and microglia-like cells now enable disease modeling using heterotopic 2D cell-cell interaction models (Abud et al., 2017; di Domenico et al., 2019).

Given the complex etiology of PD, investigating the role of spatial tissue organization, cell-cell- and cell-matrix connections is likely to be crucial in determining new mechanisms in PD pathogenesis. The possibility to differentiate stem cells into 3D organ-like structures termed *organoids* now offers a variety of opportunities to study neurodegenerative diseases (Kadoshima et al., 2013; Lancaster et al., 2013). Specifically, the patterning of organoid differentiation toward distinct brain-region specific fates, including midbrain-like organoids containing DA neurons, is of particular relevance in terms of PD (Qian et al., 2016; Smits et al., 2019).

However, despite this astonishing progress, disease modeling using human stem cells is still accompanied by a number of caveats. Line-to-line variability is a prominent challenge in identifying even subtle disease phenotypes in stem cell-derived PD models. Consequently, genome editing techniques have become highly important for the control of genetic variation as they enable the introduction of a pathogenic mutation into a control line (Soldner et al., 2016) or the correction of a mutation in a patient line (Reinhardt et al., 2013b). The development of CRISPR technology by Doudna and Charpentier (Jinek et al., 2012) has thus greatly facilitated the generation of isogenic iPSC lines, i.e., lines that have the same genetic background, differing only in the mutation of interest.

An additional pitfall of iPSC and ESC derived model system arises from the reprogramming process itself, which has been shown to reset the epigenetic landscape of the derived cells into a more embryonic-like state (Maherali et al., 2007; Guenther et al., 2010). As aging constitutes one of the major risk factors for neurodegenerative diseases, it is not surprising that age-specific epigenetic signatures emerge as potential additional drivers in their pathogenesis (Hwang et al., 2017). Transdifferentiation protocols, which allow the direct reprogramming of human fibroblasts into neurons without an intermediate stem cell state, has thus been pushed forward in order to preserve possible patient-associated epigenetic changes (Ladewig et al., 2012; Liu et al., 2013).

**Figure 1:**
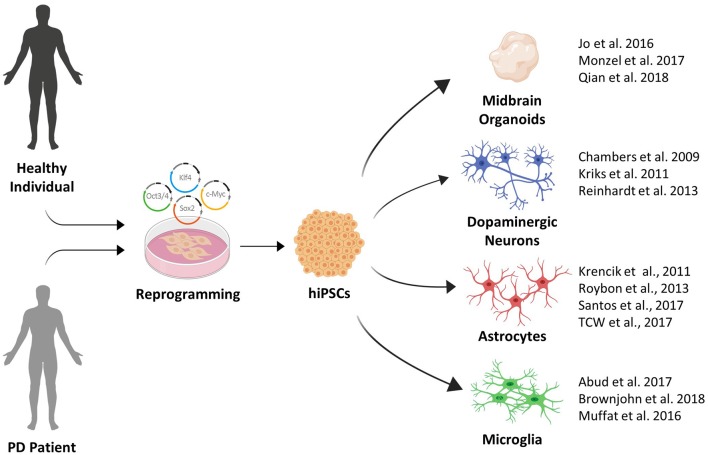
The growing induced pluripotent stem cell (iPSC) toolbox for Parkinson’s disease (PD) disease modeling.

In summary, extremely productive efforts by the stem cell field in recent years have greatly expanded the toolbox available for PD disease modeling (see Figure 1). This toolbox has been essential in identifying pathological phenotypes in human stem cell models of familial and sporadic PD. In the next section, we will provide an overview of the major phenotypes that were recently identified.

## Major Phenotypes in Human iPSC Models of PD

### Neurite Defects

**Figure 2:**
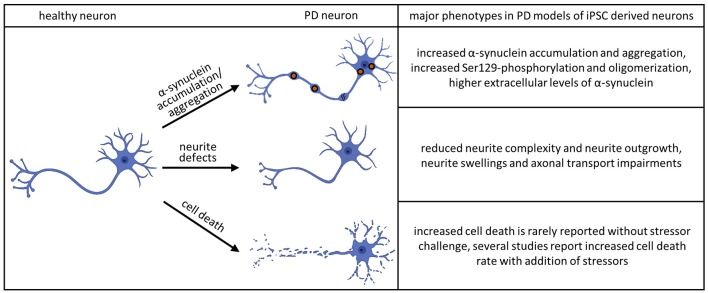
Major phenotypes observed in iPSC-derived dopaminergic (DA) neurons. The accumulation and aggregation of α-synuclein in PD models of iPSC derived neurons was shown in the following publications: Byers et al. (2011), Devine et al. (2011), Nguyen et al. (2011), Imaizumi et al. (2012), Reinhardt et al. (2013b), Ryan et al. (2013), Flierl et al. (2014), Woodard et al. (2014), Oliveira et al. (2015), Reyes et al. (2015), Chung et al. (2016), Mazzulli et al. (2016), Heman-Ackah et al. (2017), Kouroupi et al. (2017), Vasquez et al. (2017), Kim et al. (2018), Ludtmann et al. (2018), Prots et al. (2018), Hu et al. (2019), Tagliafierro et al. (2019) and Zambon et al. (2019). The phenotype of aberrant neurite morphology was described in Sánchez-Danés et al. (2012), Reinhardt et al. (2013b), Woodard et al. (2014), Schwab and Ebert (2015), Borgs et al. (2016), Lin et al. (2016), Kouroupi et al. (2017), Sommer et al. (2018) and Korecka et al. (2019). Increased cell death without additional stressors could be detected in Sánchez-Danés et al. (2012) and Bogetofte et al. (2019). Several publications detected increased cell death rate in PD models of iPSC derived neurons adding additional stressors: Byers et al. (2011), Nguyen et al. (2011), Miller et al. (2013), Reinhardt et al. (2013b), Flierl et al. (2014), Ryan et al. (2014), Shaltouki et al. (2015), Chung et al. (2016), Lin et al. (2016) and Sommer et al. (2018).

Human iPSC technology offers a unique opportunity to analyze specific neuronal structures, such as neurites, during maturation and development of pathological features. Studies employing these techniques have revealed abnormalities in iPSC-derived neurons to be one of the most consistent features across different neurodegenerative diseases. In PD pathology, changes in neurite morphology have been associated with mutations in *PARK2*, *LRRK2*, and *SNCA* (Ren et al., 2015; Lin et al., 2016; Kouroupi et al., 2017; Korecka et al., 2019; see Figure 2).

In *PARK2* iPSC derived neurons, decreased microtubule stability affects neurite complexity. It was possible to rescue neurite complexity by overexpressing parkin or by stabilizing the microtubule network using microtubule-stabilizing compounds. Long term culture of *LRRK2*^G2019S^ mDA neurons displayed decreased neurite length, as well as a number of neurites per neuron, which is further exacerbated by the treatment with 6-OHDA and Mg132 (Sánchez-Danés et al., 2012; Lin et al., 2016). *LRRK2*^G2019S^ mDA neurites are also especially susceptible to decreases in Ca^2+^ influx, as chronic treatment with thapsigargin resulted in neurite collapse (Korecka et al., 2019). A decreased neurite outgrowth rate in *LRRK2*^G2019S^ patient-derived neurons was also reported by Reinhardt et al. (2013b), who were able to restore outgrowth velocities to the level of wild-type controls using targeted gene correction. This phenotype was recapitulated by introducing the G2019S mutation into the *LRRK2* loci of a control line. The impairment in neurite outgrowth was also detectable after 5 days of differentiation. At this differentiation stage *LRRK2*^G2019S^, immature neurons display a shorter, but considerably more complex, neurite branching (Borgs et al., 2016).

Mutations in *LRRK2* also affect sensory neurons: while neurite length and complexity of sensory neurons were not affected by *LRRK2* mutations, increased neurite swellings containing cytoskeletal proteins, including tau and p-Tau, were present (Schwab and Ebert, 2015).

In addition to neurite morphology, functional parameters such as synaptic connectivity and axonal transport are impaired in PD (Kouroupi et al., 2017; Prots et al., 2018). The proportion of mobile mitochondria was reportedly increased in iPSC-derived neurons from symptomatic and presymptomatic PD patients carrying *LRRK2*^G2019S^ and *LRRK2*^R1441C^ mutations, respectively (Cooper et al., 2012). Although the mechanism behind these impairments remains elusive, the conformation, degree of aggregation and phosphorylation of α-Syn evidently has a role in the maintenance or formation of neurites (Qing et al., 2017; Prots et al., 2018).

It is important to note that mutations in *GBA* and *LRRK2* alter the levels of *SNCA* (Woodard et al., 2014; Oliveira et al., 2015; Lin et al., 2016). An interesting study carried out in monozygotic twins harboring a heterozygous GBA mutation showed displaced α-syn in the affected twin only, further suggesting that the quantity of α-Syn and its distribution along the neurons play a key role in neurite support (Woodard et al., 2014). The same study also indicated that not only the genetic predisposition but also additional factors contribute to the penetrance of PD. In this regard, studies on sporadic PD cases are especially valuable to shed light on which additional factors may alter disease onset and progression. Different cohorts of iPSC derived DA neurons from sporadic PD patients reported impairments in neurite complexity; an autologous PD Lymphocyte/iPSC-DA Neuron co-culture model for neuroinflammation, and prolonged differentiation of 2D DA neuronal cultures (Sánchez-Danés et al., 2012; Marrone et al., 2018; Sommer et al., 2018). These studies showed stress-related decreased neurite complexity in PD patients, suggesting that impairments in morphology and neurite outgrowth might be a general feature in PD pathology.

### Aggregation

The pathological hallmarks of PD are Lewy bodies and Lewy neurites, which are principally composed of aggregated α-Syn. The importance of α-Syn is further emphasized by *SNCA* mutations that have been shown to cause familial forms of parkinsonism with early-onset and fast disease progression. *SNCA* duplications or triplications and point mutations (A53T, A30P, E46K) cause PD by increasing the gene dosage or altering the biochemical properties of α-Syn (Kang et al., 2011). iPSC-derived neurons generated from PD patients or mutations introduced by CRISPR technology were able to model α-Syn aggregation as the major pathological hallmark of the disease (see Figure 2). Furthermore, iPSC derived models could replicate important mechanisms of α-Syn aggregation, such as oligomerization, phosphorylation, or secretion.

α-Syn accumulation has been demonstrated in iPSC derived PD neurons of *SNCA* duplication (Prots et al., 2018), *SNCA* triplication (Byers et al., 2011; Devine et al., 2011; Flierl et al., 2014; Oliveira et al., 2015; Reyes et al., 2015; Heman-Ackah et al., 2017; Vasquez et al., 2017) and point mutations in *SNCA* ^A53T^ (Ryan et al., 2014; Kouroupi et al., 2017), *LRKK2*^G2019S^ (Nguyen et al., 2011; Reinhardt et al., 2013b), *PARK2*^V324A^ (Imaizumi et al., 2012; Chung et al., 2016), *PINK1*^Q456X^ (Chung et al., 2016) and *GBA*^N370S^ (Woodard et al., 2014; Kim et al., 2018). A study introducing a PD-associated risk variant in an isogenic iPSC line using CRISPR technology was able to determine an enhancer element that regulates α-Syn expression (Soldner et al., 2016).

The accumulation of insoluble α-Syn, expected to be part of the aggregation cascade of α-Syn, was detected in a diverse set of genetic PD models such as *SNCA* duplication (Prots et al., 2018), *SNCA* triplication (Mazzulli et al., 2016; Ludtmann et al., 2018; Tagliafierro et al., 2019) and the point mutations *SNCA*^A53T^ (Ryan et al., 2013; Kouroupi et al., 2017) *PARK2*^V324A^ (Chung et al., 2016), *PINK1*^Q456X^ (Chung et al., 2016) and *GBA*^N370S^ (Kim et al., 2018). This reproduces aggregation as the shared disease process of PD and underlines iPSC-derived models as an adequate tool to investigate synucleinopathies on a cellular level. The higher aggregation propensity of α-Syn was detected by detergent-insoluble α-Syn (Chung et al., 2016; Mazzulli et al., 2016; Kim et al., 2018; Prots et al., 2018), as well as Thioflavin staining (Ryan et al., 2013; Kouroupi et al., 2017).

Together with the higher aggregation rate, changes such as phosphorylation and oligomerization were recapitulated in iPSC PD models. Increased Ser129-phosphorylation of α-Syn could be detected in iPSC models of *SNCA* triplication (Lin et al., 2016) *SNCA*^A53T^ point mutation (Ryan et al., 2013; Kouroupi et al., 2017; Hu et al., 2019) and the *PARK2* mutation (Ryan et al., 2013; Lin et al., 2016; Kouroupi et al., 2017). Oligomers were previously shown to be a neurotoxic α-Syn species (Winner et al., 2011). Multiple iPSC-derived *SNCA* triplication models could show an increase in α-Syn oligomers that were connected to axonal transport impairments (Prots et al., 2018) and activation of the mitochondrial transition pore (Ludtmann et al., 2018).

The main physiological α-Syn species remains controversial. Studies detected a physiological tetrameric α-Syn species, resistant to aggregation (Bartels et al., 2011; Wang et al., 2011; Dettmer et al., 2015b). A *SNCA*^A53T^ point mutation (Dettmer et al., 2015a) and *GBA*^N370S^ mutations (Kim et al., 2018) were shown to disrupt this tetrameric form and increase the monomeric α-Syn level in iPSC derived neurons.

*Braak* stated the hypothesis of spreading α-Syn pathology with the course of the disease (Braak and Braak, 2000). One route of α-Syn spreading is thought to be the secretion of α-Syn into the extracellular space. IPSC-derived models could show increased extracellular levels of α-Syn in *GBA*^N370S^ mutations (Fernandes et al., 2016) and *SNCA* triplication cases (Zambon et al., 2019). A recent study identified *LRRK2*^G2019S^ astrocytes as another source of α-Syn spreading to neurons (di Domenico et al., 2019). This study stresses the flexibility of the iPSC models to answer cell-type-specific questions, as co-cultures of control astrocytes and *LRRK2*^G2019S^ mutated neurons and vice versa could give indications about the effect of certain cell types on one another.

Recently, premature aging was introduced to iPSC models of *SNCA* triplication by extended passaging at the NPC state. These aged neurons showed a higher α-Syn aggregation rate compared to the young *SNCA* triplication neurons (Tagliafierro et al., 2019). The incorporation of aging adds an additional layer of complexity that can be modeled by iPSC-derived PD neurons. Thus, iPSC-derived models enable us to test hypotheses about how PD-causing mutations affect α-Syn species and the aggregation process.

### Cell Death

Loss of DA neurons in the SNpc is another hallmark of PD. The current hypothesis is that both genetic and environmental factors contribute to PD-linked neuronal cell death (Ball et al., 2019). This combination makes it challenging to study the disease *in vitro* since the exposure to specific environmental factors is usually difficult to trace back. *PARK2* KO derived neurons showed a decreased survival after 14 days of differentiation compared to isogenic WT lines (Shaltouki et al., 2015) and displayed higher levels of necrosis after 25 days (Bogetofte et al., 2019). However, besides these studies, neuronal cell death is rarely reported in iPSC-derived neuronal cultures without a prior stressor challenge (see Figure 2). One of the best-studied mutations in PD is the *LRRK2*^G2019S^. However, in the majority of studies, no changes in cell viability were reported under basal conditions. Sánchez-Danés et al. (2012) were able to find increased cell death occurring in sporadic and familial cases of *LRRK2*- related PD *in vitro* after prolonged differentiation. This can be attributed to the increased maturity of the neuronal culture. Longer neuronal cultures can also be achieved by generating brain organoids. However, to date only two studies have modeled PD using PD-patient derived midbrain organoids (Kim et al., 2019; Smits et al., 2019) and cell death was reported in only one of these (Kim et al., 2019).

One possible explanation for the lack of cell death in iPSC-derived cultures under basal conditions may be the use of well-adjusted cell culture media and supplements, which were specially developed to support neuronal survival and counteract oxidative stress. In addition, for neuronal differentiation, this media is usually supplemented with additional neurotrophic factors, including BDNF and GDNF (Björklund et al., 1997; Zuccato and Cattaneo, 2009). Thus, this media combination may mask some of the neurodegenerative phenotypes we wish to model.

A second explanation for the lack of cell death may be the loss of age-related markers, which inevitably occurs during the process of reprogramming (Marion et al., 2009). These age-related traits that are lost are not regained even in longer *in vitro* maturation and consequently, these neurons still do not faithfully mimic the age-related traits of an e.g., 50-year-old PD patient neuron. Transdifferentiation might be an option to retain age-related epigenetic traits for future experiments (Mertens et al., 2015).

Despite these drawbacks, such neuronal cultures are especially valuable when investigating early transcriptomic and proteomic pathological changes, which may ultimately lead to activation of apoptotic pathways. Transcriptomic analysis of *SNCA* triplication in cortical neurons suggest that endoplasmatic reticulum stress followed by activation of the IRE1a/XBP arm of the unfolded protein response might be responsible for cell death in PD (Heman-Ackah et al., 2017). Microarray analysis of DA neurons derived from two patients with A53T mutation in *SNCA* was associated with S-nitrosylation of the anti-apoptotic transcription factor MEF2C contributing to mitochondrial dysfunction (Ryan et al., 2013). Moreover, increase in oxidative stress and inflammation was associated with induction of the necroptotic pathway in OPA-1 derived NPCs (Iannielli et al., 2018). An autologous mDA-lymphocyte co-culture suggested activation of the NFkB pathway, bringing neuroinflammation into the spotlight (Sommer et al., 2018). Finally, proteomic analysis of *PARK2* KO iPSC-derived neurons and isogenic lines used as controls identified dysregulation of proteins involved in oxidative stress defense, mitochondria respiration, cell cycle control and cell viability (Bogetofte et al., 2019). Further, “omic” studies may shed some light on early-associated PD-specific dysregulated pathways.

### Stressor Response

A set of publications has been able to link cell death pathways to dysregulated upstream cellular functions in PD patients’ cells. These studies indicate a decrease in viability upon exposure to stressors that challenge these dysregulated functions. An example of these studies identifying and then challenging the dysregulated pathway is published by Nguyen et al. (2011). They first showed increased expression of genes involved in oxidative stress response in iPSC-derived *LRRK2*^G2019S^ patient neurons. When adding the oxidative stressor H_2_O_2_, the patients’ derived DA neurons revealed a higher cell death rate (Wang et al., 2011). Thus, this study demonstrated that iPSC models are a powerful tool to study the environmental component *in vitro*. As previously mentioned, both environmental and genetic factors contribute to the current hypothesis of PD pathogenesis. Oxidative stress, mitochondrial dysfunction, proteasome inhibition, and aging factors have all been identified as playing a role in PD. The following four paragraphs describe publications that have combined these factors with genetic iPSC PD models.

Mitochondrial dysfunction is a core pathomechanism of PD (Franco-Iborra et al., 2018). This is underlined by the environmental risk factor rotenone, which inhibits the complex I of the mitochondrial electron transport chain and is epidemiologically linked to PD (Tanner et al., 2011). Another mitochondrial stressor inhibiting mitochondrial complex I, MPTP, also causes PD symptoms within days of exposure (Langston, 2017). An increased susceptibility towards rotenone (Cooper et al., 2012; Ryan et al., 2013; Flierl et al., 2014; Mittal et al., 2017; Tabata et al., 2018) and MPTP (Kim et al., 2019) was also determined in neurons and organoids from iPSC-derived PD patients’. Carbonyl cyanide m-chlorophenyl hydrazine (CCCP) decreases the mitochondrial membrane potential and *PINK1* and *PARK2* iPSC-derived DA neurons were more prone to cell death after treatment with CCCP (Chung et al., 2016).

Oxidative stress is one of the major biochemical processes thought to impair the vulnerability of DA neurons in PD. Pesticides and heavy metals (Tanner et al., 2011; Farina et al., 2013), as well as PD-causing mutations (e.g., *PARK2*, *PINK1*, *SNCA*, *LRRK2*; Blesa et al., 2015), have been shown to induce oxidative stress. Several factors known to induce oxidative stress are used in iPSC PD studies, such as the pesticide paraquat, the synthetic dopamine analog 6-hydroxydopamine (6-OHDA) and the reactive oxygen species H_2_O_2_. Paraquat is epidemiologically linked to PD (Tanner et al., 2011) and increased susceptibility to this pesticide could be determined in iPSC PD neurons (Ryan et al., 2013; Lin et al., 2016). 6-OHDA is a synthetic neurotoxin, taken up *via* the dopamine transporter and selectively damaging DA neurons due to ROS generation (Hernandez-Baltazar et al., 2017). An increased vulnerability of PD patients’ derived neurons towards 6-OHDA has been determined (Nguyen et al., 2011; Lin et al., 2016). Adding the reactive oxygen species H_2_O_2_ to *LRRK2*^G2019S^ (Nguyen et al., 2011), *LRRK2*^I2020T^ (Ohta et al., 2015) or *SNCA* triplication neurons (Byers et al., 2011), revealed a higher vulnerability of cells carrying PD-causing mutations towards oxidative stress. There is evidence linking heavy metal exposure to the development of PD, thought to be at least partly due to the generation of oxidative stress (Ball et al., 2019). An increased susceptibility towards the heavy metals copper (Aboud et al., 2015), cadmium (Aboud et al., 2015), and manganese (Aboud et al., 2012) were determined in iPSC-derived PD patients’ neurons.

The proteasome system, which plays a central role in degrading dysfunctional proteins, has been shown to be impaired in the postmortem substantia nigra (SN) of PD patients (Bentea et al., 2017). Exposure to the proteasomal inhibitor carbobenzoxyl-L-leucyl-L-leucyl-L-leucine (MG-132) determined a higher susceptibility in PD patients’ cells (Nguyen et al., 2011; Lin et al., 2016).

Aging is a major risk factor for PD. Reprogramming has been shown to rejuvenate the cells by resetting aging markers, e.g., mitochondrial ROS, nuclear morphology markers and DNA damage markers (Miller et al., 2013). Aging is known to drive major pathomechanisms of PD, e.g., mitochondrial and oxidative stress (Rodriguez et al., 2015). The addition of aging factors can provide a way of determining higher susceptibility within iPSC models of genetic PD that is compensated for during the rejuvenation in the iPSC model. Studies have so far added a variety of aging factors together with different PD-causing mutations, all of which resulted in a higher susceptibility. Several strategies have been applied to induce premature aging, e.g., telomerase inhibition (Vera et al., 2016), progerin expression (Miller et al., 2013) and higher NPC passaging (Tagliafierro et al., 2019). The susceptibility towards the aging factors was not specific to a certain mutation, as shown for *PARK2*^V324A^ (Miller et al., 2013) *PINK1*^Q456X^ (Miller et al., 2013; Vera et al., 2016) and SNCA triplication cases (Miller et al., 2013; Vera et al., 2016; Oh, 2019).

IPSC technology and epidemiology can both provide valuable insights into Parkinson disease. The addition of environmental risk factors to iPSC-derived models generates PD models that can investigate the combinatorial effects of genes and the environment. This allows a deeper insight into the interactions and dependencies between the affected cellular processes critical to the cell to maintain homeostasis. This can help to determine the key cellular pathways challenged by multiple exposures occurring over a lifetime, recently summarized by the concept of the “neuroexposome.” These combinatorial effects may be of particular significance when investigating genetic risk factors e.g., SNPs identified in genome-wide association (GWAS) studies. While genetic risk factors increase the risk for developing PD, they are not causative and depend on the presence of other risk factors, including environmental factors. This interplay of genetic risk factors that render DA neurons more susceptible to environmental factors takes place in sporadic PD. Indeed, our lab was able to identify an increased vulnerability of iPSC-derived, sporadic PD patients’ neurons towards interleukin 17. The cell death rate of sporadic PD patients’ cells was equal to cells from healthy individuals in unstressed conditions, but significantly higher when exposed to IL-17 secreting T helper 17 cells (Sommer et al., 2018).

Neurodegenerative phenotypes in PD patients’ neurons become more evident with the addition of cellular stressors. Yet the comparability of different studies remains problematic, as differentiation protocols, maturation time as well as stressor dosage and duration tend to vary between different studies.

## Modeling Cell-Cell Interactions in PD Using Human iPSC-Derived Models

As mentioned above, mounting evidence indicates that neurodegeneration in PD is not a neuron-autonomous process, but rather the result of a complex detrimental interplay between a variety of different cell types (Hirsch et al., 2013). Recent advances in the differentiation of human iPSCs and ESCs have now expanded the toolbox available for studying PD *via* 2D co-culture models as well as 3D organoid models. In the following section, we discuss recent findings of studies using these models, highlighting specific cell-cell interactions as well as organoid models. Moreover, we also address new possibilities for future investigations of cell-cell interactions in PD.

### Neuron—Astrocyte

Although the majority of existing studies on PD aimed to investigate neuronal function, there is now increasing evidence that astrocytes play an important role in PD pathogenesis. Recent transcriptomic analyses using human and rodent CNS-resident cells have shown that a variety of PD associated genes are expressed in human astrocytes to a similar, and sometimes even larger extent, compared to neurons (Zhang et al., 2016). Astrocyte-specific functions have been identified for a variety of genes known to be causative for PD, including *DJ-1, SNCA, PARK1, PARK2, PARK7, PLA2G6*, and *ATP13A2*. These functions include regulation of astrocyte neurotrophic properties and their response to inflammatory stimuli, as well as astrocytic glutamate transport and mitochondrial function (reviewed extensively in Booth et al., 2017). Most of these studies were conducted in both rodent models and in post-mortem human brain tissue. While stem-cell-derived technologies now open up many possibilities to study astrocyte function in a human system (Krencik and Zhang, 2011; Roybon et al., 2013; Santos et al., 2017; Tcw et al., 2017; Perriot et al., 2018), studies on astrocyte function in PD using iPSC- or ES-derived cellular systems remain scarce.

Using a co-culture system of iPSC-derived neural progenitor cells and astrocytes, astrocytes exerted a protective effect. Interestingly, while treatment with mitochondrial stressors rotenone and potassium cyanide led to a significant decrease in NPC maturation into DA neurons, co-culture with iPSC-derived astrocytes rescued differentiation deficits and mitochondrial dysfunction in iPSC-derived DA neurons (Du et al., 2018). These results clearly highlight the important role of astrocytes in regulating mitochondrial homeostasis of DA neurons under stress conditions. However, it has yet to be determined how this protective effect during differentiation may be translated into a PD disease context, given the fact that neurodegeneration in PD sets in after maturation of DA neurons. Moreover, it is not clear whether PD-causing pathogenic variants may lead to a disturbance of these protective astrocyte functions during differentiation.

A recent study by di Domenico et al. (2019) made use of a neuron-astrocyte co-culture approach to study the effect of a PD-causing *LRRK2* mutation on neuron-astrocyte crosstalk (G2019S). Remarkably, the co-culture of control iPSC-derived mDA neurons with patient-derived astrocytes harboring the LRRK2 G2019S mutation exerted a neurodegenerative phenotype, while co-culture with control astrocytes did not result in this effect. The neurodegenerative phenotype was characterized by decreased cell survival and morphological alterations in mDA neurons, as well as an accumulation of α-Syn in mDA neurons and mutant astrocytes. Activation of chaperone-mediated autophagy was, at least in part, able to rescue these effects (di Domenico et al., 2019).

These results emphasize the dual role astrocytes may play during PD pathogenesis. On the one hand, the loss of astrocytic neurotrophic and neuroprotective properties might be an important driver of PD pathogenesis. On the other hand, astrocytes could gain neurotoxic abilities upon progression of the disease and may themselves release neurotoxic factors. As an inadequate response and higher vulnerability to environmental stressors are possible causes for sporadic PD, modeling sporadic PD *via* neuron-astrocyte interactions upon different stressor conditions may be of particular interest. Interestingly, recent studies showed that PD patient brain samples harbor an increased number of senescent astrocytes (Chinta et al., 2018). Whether these senescent astrocytes contribute to neurodegeneration *via* the loss of their neuroprotective function or the release of neurotoxic factors remains to be elucidated. Stem-cell derived co-culture systems may provide a valuable platform in deciphering the role of these astrocytes in PD pathogenesis. However, it may be difficult to model the late-stage effects of an aberrant astrocyte-neuron interaction in PD using iPS derived 2D co-culture approaches due to the fetal identity of iPS derived neurons and astrocytes. The addition of stressors inducing aging effects may thus be needed in the co-culture set up in order to gain insights into the contribution of astrocytes to later time points of PD pathogenesis.

### Neuron—Immune Cells

Microglia represent the resident macrophage population in the central nervous system (CNS). In response to a variety of disease-associated stimuli, including microbes or even unfolded and aggregated proteins like α-Syn oligomers and fibrils, microglia can become activated and initiate an inflammatory response in CNS (Wolf et al., 2017). Several studies have provided direct evidence for a microglia-mediated inflammatory response in neurodegenerative diseases including PD (reviewed in Song and Colonna, 2018). In post-mortem studies of PD patient brains, as well as in living patients, microglia activation has been demonstrated (Hirsch et al., 2003; McGeer and McGeer, 2008). Interestingly, epidemiological studies were able to show that the risk of PD is reduced in patients receiving long-term treatment with certain nonsteroidal anti-inflammatory medications, supporting the idea that inflammatory pathways contribute to PD progression (Bartels et al., 2010; Rees et al., 2011). Until now, the vast majority of studies on microglial function in health and disease have relied on rodent models. As recent studies indicated major transcriptomic differences between human and rodent microglia (Butovsky et al., 2014; Gosselin et al., 2017), the investigation of microglial function in a human system is urgently required to delineate the mechanisms and functional consequences of microglia activation in human neurodegenerative diseases. Protocols generating microglia-like cells (iMGLs) from iPSCs may provide a valuable resource in characterizing human microglial function. As microglia originate from hematopoietic stem cells of the yolk sac (Ginhoux et al., 2010), microglia differentiation differs extensively from differentiation procedures of neuroectoderm-derived cells such as neurons, oligodendrocytes, and astrocytes. Muffat et al. (2016) were the first to report a protocol for microglia differentiation based on the induction of cystic embryoid bodies resembling yolk-sac-like cells and subsequent maturation with colony-stimulating factor 1 and IL-34. A feeder and serum-free differentiation method, which exhibited good scalability and high purity were first described by Abud et al. (2017). They made use a two-step differentiation in which iPSCs were first differentiated into hematopoietic stem/progenitor cells (HSPCs) and afterward matured into iMGLs.

Thus far, existing literature delineating the role of microglia in neurodegenerative diseases using iMGLs remains sparse. Nevertheless, a number of studies in other neurodegenerative diseases point toward the possible impact of iMGLs. A study by Lin et al. (2016) showed morphological alterations and a reduced capacity of extracellular amyloid-beta clearance in iPSC-derived microglia-like cells carrying the ApoE4 risk variant for Alzheimer’s disease compared to controls. Using iPSC cells from patients carrying a disease-causing variant of the TREM2 protein, another study was able to show that TREM2 deficient microglia show specific deficits in phagocytosis of apoptotic cells (Garcia-Reitboeck et al., 2018). Like astrocytes, microglia may play a dual role in PD pathophysiology by exerting both a protective and detrimental effect dependent on the disease-state.

A number of different concepts and hypotheses for the role of microglia in neurodegeneration have been developed in an effort to summarize the existing data. The concept of “microglia-priming” assumes that microglia exhibit an inherently aberrant response to disease-associated proinflammatory stimuli in neurodegeneration—potentially initiating and exacerbating the course of the disease (Perry and Holmes, 2014). Another possibility is that microglia respond adequately to neuroinflammatory stimuli before disease onset, but then undergo a phenotypic switch towards detrimental “degeneration-associated-microglia” as a result of accumulating inflammatory stimuli and aging effects (Song and Colonna, 2018). An investigation of the human microglia response to different PD associated stimuli may shed light on these different concepts in Parkinson disease. However, it has to be taken into account that microglial identity and function strongly relies on the presence of paracrine and juxtacrine cues provided by surrounding cells of the CNS including neurons (Abud et al., 2017; Gosselin et al., 2017; Hasselmann et al., 2019). Thus, a co-culture of iMGLs with iPSC-derived mDA neurons, in particular, may represent a valuable system in deciphering the effect of microglia on DA neurons under steady-state and stress conditions in a more physiological environment. As iMGLs and iPSC-derived mDA neurons have been shown to more closely resemble their fetal cellular counterparts, an iMGL-mDA neuron co-culture may be of specific value for the investigation of a possible “primed microglia” phenotype. Nevertheless, more sophisticated 2D co-culture systems also including other glial cells as transducers or even more complex 3D culture systems may be needed to more closely model *in vivo* human microglial function in a PD disease context (see “Organoids” section).

The concept of neuroinflammation in PD has long been principally attributed to innate immune cells of the CNS, like microglia (Muffat et al., 2016). However, studies that demonstrated the activation of T lymphocytes by α-Syn, as well as T-cell subset alterations in blood samples of PD patients, raised the possibility of a peripheral immunity involvement in PD pathogenesis (Baba et al., 2005; Stevens et al., 2012; Sulzer et al., 2017). Animal models have provided some functional evidence for a T-lymphocyte involvement in PD pathology (Brochard et al., 2009; Reynolds et al., 2010; Sommer et al., 2016). In addition, a recent study by Sommer et al. (2018) made use of a co-culture system between T-lymphocytes and iPSC-derived midbrain neurons to enumerate the role of T-cells in sporadic PD. Strikingly, the autologous co-culture of patient midbrain neurons with patient IL-17 producing T-cells leads to increased cell death in neurons compared to a non-autologous co-culture. This effect was dependent on IL-17 receptor signaling, as blockage of IL-17 or IL-17 receptor rescued neuronal death (Sommer et al., 2018). These results emphasize the possible role of adaptive immune cells in PD pathogenesis. Adding microglia to a co-culture system of T-cells and neurons may thus provide a means of determining how the interplay between adaptive and innate immunity shapes PD pathogenesis.

### Organoids

Generation of human iPSC-derived DA neurons from PD patients could recapitulate certain disease phenotypes such as neurite pathology or neuronal cell death. Although the co-culture of DA neurons and different cell types such as astrocytes or microglia may bring the *in vitro* model closer to the *in vivo* situation, the complex organization of the human brain remains a major challenge. One of the difficulties in modeling late-onset neurodegenerative diseases like PD is the immaturity of human iPSC-derived DA neurons *in vitro*. Moreover, current 2D culture systems lack cell-cell interactions of neurons and non-neuronal cells such as glia cells in a spatially organized environment.

Recent advances in the generation of 3D cell culture systems, so-called organoids, open up new possibilities for iPSC-based disease modeling of PD. Differentiation protocols for human brain organoids make use of the intrinsic self-organization capability of pluripotent stem cells. Aggregation of single-cell iPSCs into embryoid bodies mimics the organization of the three germ layers during embryonic development. Culturing in minimal medium enables the differentiation of the outer layer to ectodermal fate. Embedding of these structures in extracellular matrices leads to a further outgrowth of neuroepithelium by serving as a supporting scaffold and induction of the apical-basal polarity as it is seen for the neural tube during embryonic neurodevelopment. Consequently, brain-region specific organoids offer enormous possibilities for long-term disease modeling of human neurodegeneration in specialized brain regions, such as the SN in PD.

Similar to the 2D differentiation of human iPSC-derived DA neurons, human midbrain organoids (hMOs) containing DA neurons are generated by patterning of iPSCs to midbrain fate. Either human iPSCs (Jo et al., 2016; Qian et al., 2016; Kim et al., 2019) or already patterned neural stem cells (Monzel et al., 2017; Smits et al., 2019) are aggregated as single cells to form hMOs.

Midbrain specific differentiation approaches led to radially organized midbrain organoids consisting of a proliferative ventral zone, an intermediate zone, and an outer neuronal layer. After around 2 months, the hMOs express the respective midbrain markers (e.g., FOXA2, dopamine transporter DAT) and contain 54%–65% TH-positive DA neurons. Electrophysiological analysis by whole-cell patch clamping and multielectrode arrays (MEA) revealed functional neurons and synchronized neuronal networks. Moreover, the accumulation of neuromelanin, a byproduct of dopamine metabolism in 2 months old hMOs, represents an intriguing line of inquiry for disease modeling.

To date, only two studies describe disease modeling of PD using hMOs (Kim et al., 2019; Smits et al., 2019). In both cases, the focus was on a specific *LRRK2* mutation (G2019S). *LRRK2*^G2019S^ hMOs have deficits as indicated by a decreased number of TH-positive neurons and a reduction of neuronal complexity. In addition, increased MPTP-induced cell death of DA neurons in mutant organoids and abnormal localization of phosphorylated α-Syn was present. Inhibition of LRRK2 kinase activity resulted in a reduction of DA cell death and a reduction of phosphorylated α-Syn.

These results indicate that hMOs can indeed recapitulate aspects of PD pathology that have been already shown in 2D models. However, human brain organoids lack some glial cell types like oligodendrocytes or microglia (Quadrato et al., 2017; Sloan et al., 2017). As a result, co-culture models of midbrain organoids and non-neuronal cell types are required. Recent transcriptomic studies on human microglia were able to determine that their transcriptional identity is heavily dependent on intercellular, three-dimensional cues (Gosselin et al., 2017). It will be challenging to establish applicable 3D co-culture models due to the appropriate timing of differentiation and media compositions to introduce one or more additional cell types into the organoids. Moreover, activation of apoptosis within larger organoids due to lack of oxygen and nutrients (Mansour et al., 2018) might interfere with PD models focusing on oxidative stress.

It is suspected that the leakiness of the blood-brain-barrier (BBB) may lead to the observed infiltration of peripheral immune cells (Sommer et al., 2018). Consequently, 3D culture conditions need to be further optimized for the co-culture of hMOs and, for example, patient-derived blood lymphocytes. Nonetheless, one needs to consider that stem cell-based models still resemble fetal stages of neurodevelopment and that even premature aging using substances like progerin creates an artificial model of age-related phenotypes as seen in PD.

## Outlook

Since the first report describing successful reprogramming of human fibroblasts into iPSCs over a decade ago, recent progress has underscored the importance of iPSCs as a powerful tool to model neurodegenerative diseases, including PD, in a human system. As a pre-requisite, various iPSC-differentiation protocols now enable the generation of DA neurons, astrocytes, and microglia as well as complex three-dimensional neuronal structures, termed organoids. Future innovation and optimization of differentiation protocols should be able to accelerate research progress in this field even further.

**Figure 3:**
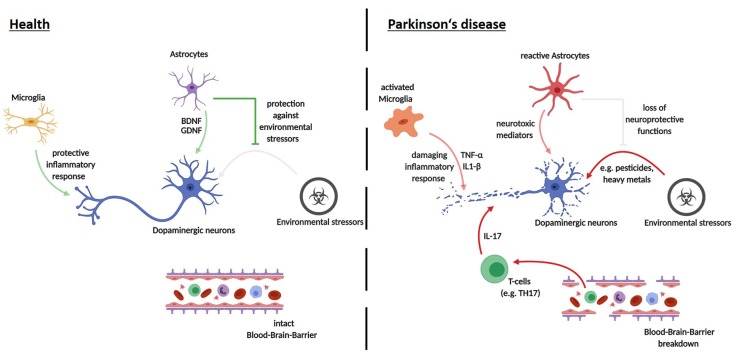
Cell-cell interactions in the pathogenesis of PD—recent advances using iPSC-derived disease modeling. Opaque arrows depict recent findings made using iPSC-derived cell-cell interaction models; pale arrows show mechanisms that still need investigation *via* iPSC-derived human disease modeling.

It should be noted that while studies on iPSC-derived midbrain dopaminergic (mDA) neurons in isolated culture systems were able to recapitulate major pathological hallmarks of PD, including neurite defects and aggregation of α-Syn, they found very little cell death. The addition of further stressors appears to be an essential step in observing cell death in iPSC-derived model systems of PD (see Figure 2). These stressors have been shown to include toxins, aging effects and the addition of other cell types to the culture system. Major reasons for limited pathology in PD models are that cultivation media are optimized to prevent neurodegeneration, the iPSC derived neural models mimic a fetal developmental stage and that major epigenetic traits are lost during reprogramming. Given the complex etiology of the neurodegenerative process in PD, co-culture approaches of different neural cell types provide unique opportunities for future disease modeling (depicted in Figure 3).

## Author Contributions

BW, KS, JL, TR and JK wrote and critically discussed this manuscript. BW, JK, TR, JL and KS generated the figures, revised the manuscript and approved the final manuscript.

## Conflict of Interest

The authors declare that the research was conducted in the absence of any commercial or financial relationships that could be construed as a potential conflict of interest.
